# OKEN: A Supervised Evolutionary Optimizable Dimensionality Reduction Framework for Whole Slide Image Classification

**DOI:** 10.3390/bioengineering12070733

**Published:** 2025-07-04

**Authors:** Soroush Oskouei, André Pedersen, Marit Valla, Vibeke Grotnes Dale, Sissel Gyrid Freim Wahl, Mats Dehli Haugum, Thomas Langø, Maria Paula Ramnefjell, Lars Andreas Akslen, Gabriel Kiss, Hanne Sorger

**Affiliations:** 1 Department of Circulation and Medical Imaging, Norwegian University of Science and Technology (NTNU), NO-7491 Trondheim, Norway; 2 Clinic of Medicine, Levanger Hospital, Nord-Trøndelag Health Trust, NO-7600 Levanger, Norway; 3 Application Solutions, Sopra Steria, NO-7010 Trondheim, Norway; 4 Department of Clinical and Molecular Medicine, Norwegian University of Science and Technology (NTNU), NO-7491 Trondheim, Norway; 5 Clinic of Laboratory Medicine, St. Olavs Hospital, Trondheim University Hospital, NO-7030 Trondheim, Norway; 6 Department of Pathology, St. Olavs Hospital, Trondheim University Hospital, NO-7030 Trondheim, Norway; 7 Department of Health Research, SINTEF Digital, NO-7465 Trondheim, Norway; 8 Center for Innovation, Medical Devices, and Technology, St. Olavs Hospital, Trondheim University Hospital, NO-7030 Trondheim, Norway; 9 Department of Clinical Medicine, Centre for Cancer Biomarkers (CCBIO), University of Bergen, NO-5007 Bergen, Norway; 10 Department of Pathology, Haukeland University Hospital, NO-5020 Bergen, Norway; 11 Department of Computer Science, Norwegian University of Science and Technology (NTNU), NO-7491 Trondheim, Norway

**Keywords:** lung cancer, digital pathology, deep learning, dimensionality reduction, evolutionary algorithm

## Abstract

Classification of lung cancer subtypes is a critical clinical step; however, relying solely on H&E-stained histopathology images can pose challenges, and additional immunohistochemical analysis is sometimes required for definitive subtyping. Digital pathology facilitates the use of artificial intelligence for automatic classification of digital tissue slides. Automatic classification of Whole Slide Images (WSIs) typically involves extracting features from patches obtained from them. The aim of this study was to develop a WSI classification framework utilizing an optimizable kernel to encode features from each patch from a WSI into a desirable and adjustable latent space using an evolutionary algorithm. The encoded data can then be used for classification and segmentation while being computationally more efficient. Our proposed framework is compared with a state-of-the-art model, Vim4Path, on an internal and external dataset of lung cancer WSIs. The proposed model outperforms Vim-S16 in accuracy and F_1_ score at both ×2.5 and ×10 magnification levels on the internal test set, with the highest accuracy (0.833) and F_1_ score (0.721) at ×2.5. On the external test set, Vim-S16 at ×10 achieves the highest accuracy (0.732), whereas OKEN-DenseNet121 at ×2.5 has the best F_1_ score (0.772). In future work, finding a dynamic way to tune the output dimensions of the evolutionary algorithm would be of value.

## 1. Introduction

Lung Adenocarcinoma (AC) and Squamous Cell Carcinoma (SCC) are the two main histological subtypes of Non-Small Cell Lung Cancer (NSCLC). The discrimination of these entities is clinically important because of their distinct biological behaviors, treatment responses, and prognoses [1,2]. While the histopathological classification of these subtypes is straightforward in many cases, poorly differentiated non-small cell lung carcinomas may pose challenges for pathologists. These cases often require immunohistochemistry (IHC) for correct subclassification [3]. An efficient and accurate automated classification system could be an addition to IHC and, thus, reduce diagnostic time and effort, particularly in ambiguous cases [4]. This underlines the importance of developing computational models capable of classifying tumor subtypes directly from Hematoxylin and Eosin (HE)-stained Whole Slide Images (WSIs) [4,5].

WSIs are enormous, containing tens of millions of pixels, which prevents their direct use in neural networks with current customary technology. To address this challenge, several strategies are employed: using downsampled versions, selecting specific regions, dividing the image into patches for individual processing, dividing WSIs into clusters, instance sampling, or even combinations of these methods [6,7,8,9].

During the past decade, computational approaches to WSI classification in digital pathology have advanced rapidly, accelerated by innovations in deep learning [10,11]. Convolutional Neural Networks (CNNs) employ convolution to capture morphological features, while Transformer-based models—specifically, Vision Transformers (ViTs)—introduce self-attention mechanisms that can effectively handle the vast spatial heterogeneity in histopathological data [12,13,14]. Although ViTs have shown promising results, they can be data-intensive and computationally expensive for large-scale images [15].

To improve the performance of ViTs, several specialized architectures have been developed. Pyramid Vision Transformers and Swin Transformers adopt multi-scale representations to handle high-resolution inputs more efficiently [16,17]. Other architectures, such as SegFormer and UNETR, focus on tasks like semantic segmentation and 3D medical image analysis [18,19]. Meanwhile, state-space models have also gained attention for their ability to model long-range dependencies in linear time [20,21]. Mamba and its vision-specific variant, Vision Mamba (Vim), exemplify this trend by harnessing state-space models for efficient high-dimensional data processing [22,23]. Despite these innovations, simpler CNN-based pipelines sometimes remain competitive [24,25,26].

Due to the large image size of WSIs, it is common to divide them into smaller image patches and process each individually. These patch-wise approaches typically aggregate patch features via simple operations. An example is DeepGrade, where several patch-wise models were trained at different magnification levels and aggregated to produce the final WSI-level prediction [27].

A challenge with patch-wise-only designs is that they lack global information during model training. To counteract this challenge, Multiple-Instance Learning (MIL) was proposed, in which each WSI is viewed as a “bag” of patches and the model is trained to perform bag-wise classification [28,29]. Along with MIL, attention mechanisms employed in methods such as CLustering-constrained Attention Multiple-instance learning (CLAM) detect important patches essential for classification, highlighting relevant regions in the tissue [30]. Other frameworks apply graph neural networks, where each patch becomes a graph node connected via edges that represent spatial or content-based relationships, allowing the model to capture broader context by encoding these interactions.

Recent methods have introduced more sophisticated designs to integrate holistic information. This is especially applicable for WSI classification, where both the local and global tissue context must be captured. Self-supervised learning techniques such as DINO-enable models to learn patch embeddings from unlabeled data, improving consequential performance when fine-tuned on classification tasks [31]. Hierarchical Transformers like HIPT stack Transformers at different resolution levels to integrate both detailed and global context [32]. Multi-magnification pipelines further extended this idea, combining high- and low-resolution patches.

Nasiri-Sarvi et al. employed Vim for histopathological WSI classification and incorporated positional encoding interpolation [33]. Although ViTs excel at modeling global contexts within images, Vim attempts to capture both local and global features more efficiently. This design is particularly relevant for WSI classification, given the massive scale and heterogeneity of histology slides. Nevertheless, the large size and heterogeneity of WSI data pose substantial hurdles—both in terms of computational overhead and in preserving meaningful class separability for clinical interpretation.

Despite advances in applying ViT and Vim to tasks like WSI classification, it is still challenging to keep computations efficient while maintaining clear class separability. Moreover, current dimensionality reduction techniques often do not aim for class separability, leading to less effective performance for classification tasks. This issue is especially significant in WSI analysis, where the data are very large and diverse, making robust dimensionality reduction valuable for more explainable results and faster computations.

In this paper, we present an Optimizable Kernel Embedded Network (OKEN) framework for classification of lung AC and SCC and reduce the computational cost by utilizing supervised dimensionality reduction. We propose an Evolutionary Algorithm (EA) that evolves a transformation matrix to reduce feature dimensionality while inducing class separability, measured through the silhouette score on known labels. This strategy aims to reduce the information entropy in high-dimensional data, making subsequent classification faster and potentially more accurate.

Our contributions include the following:

- A novel supervised dimensionality reduction of features utilizing an evolutionary algorithm;
- Integration of this technique into a new open framework (OKEN), facilitating its adaptation to various datasets and applications;
- A study to measure the impact of key components within the proposed framework and understand how each affects classification performance.

## 2. Materials and Methods

### 2.1. Datasets

In this study, we used tissue samples from two NSCLCs cohorts: a cohort from the regional research biobank in central Norway (Biobank1^®^) and the Haukeland University Lung Cancer (HULC) cohort. The Biobank1 cohort contains histopathological, cytological, biomarker, and longitudinal clinical follow-up data from patients with suspected lung cancer in Central Norway since 2006. This dataset includes diagnostic tumor biopsies and sections from surgical lung cancer specimens.

The HULC cohort consists of 438 patients with NSCLC who underwent surgical resection at Haukeland University Hospital in Bergen, Norway, between 1993 and 2010. A total of 87 cases from this cohort were included in the study. In both cohorts, 4 µm thick tissue sections were cut from formalin-fixed, paraffin-embedded tissue blocks. The sections were then stained with HE and scanned at ×40 magnification using Olympus VS120-S5 and VS200-ASW scanners (Olympus Soft Imaging Solutions GmbH, Munster, Germany). The staining protocol included deparaffinization, rehydration, hematoxylin staining, sequential rinsing, eosin counterstaining, dehydration, immersion in TissueClear, and air-drying [34].

**Figure 1:**
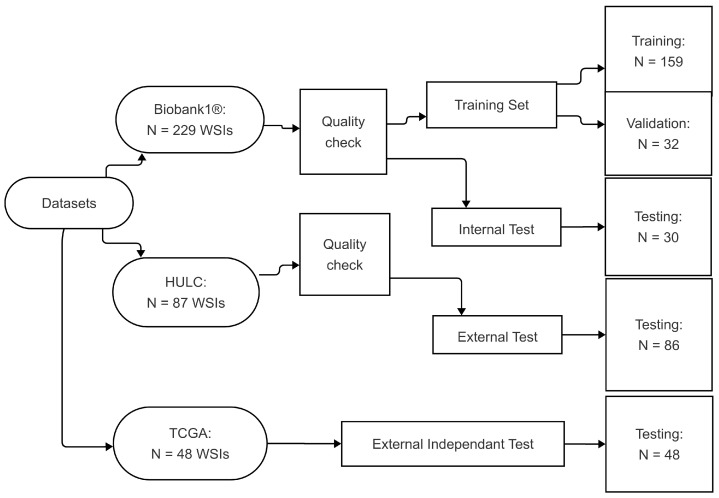
Visualization of data split and distribution. The diagram was created using Mermaid [37]. Abbreviations: HULC: Haukeland University Hospital Lung Cancer; WSIs: whole slide images; TCGA: The Cancer Genome Atlas.

A total of 229 WSIs from the Biobank1 cohort and 87 WSIs from the HULC cohort were evaluated. After excluding blurry images and those with artifacts or lacking sufficient amounts of tumor tissue, 221 WSIs from Biobank1 and 86 from HULC remained. WSIs from Biobank1 were randomly divided into subsets of 159 for training, 32 for validation, and 30 for internal testing. The HULC dataset was exclusively used as an external test set (Figure 1). The distribution of histological subtypes across the datasets is summarized in Table 1.

The Cancer Genome Atlas (TCGA) data portal presents data from 33 different cancer types and includes datasets containing WSIs [35]. In this study, we used two datasets from TCGA (LUAD and LUSC) that include histological images of HE-stained histopathological sections from patients with AC and SCC [35,36]. To test the generalizability of the proposed model, 48 images were randomly selected from the mentioned datasets with a balanced class division.

### 2.2. Preprocessing

In the presented preprocessing scheme, WSIs were first converted to a tiled pyramidal TIFF format using vsi2tif to allow for compatibility with commonly used libraries for the reading of WSIs such as OpenSlide and cuCIM  [38,39,40].

Tissue segmentation of each WSI was performed by extracting a low-resolution thumbnail of the WSI, extracting the image gradient using the Sobel operator, applying Otsu for dynamic thresholding, and using morphological opening and hole filling to refine the produced binary mask [41,42,43].

To implement the dimensionality reduction optimization algorithm, patches were extracted by applying a sliding window technique, retaining patches using two criteria: first, those containing more than 10% tissue and, second, those presenting more than 50% tumor likelihood, which was assessed with a patch-wise tumor classifier. The classifier is a variant of the patch-wise classifier used in DRU-Net [43]. This variant is a fused CNN-based model combining truncated ResNet101-V2 and DenseNet201 [44,45]. This classifier model assigns probabilities to patches, indicating tumor or non-tumor tissue. Model training was conducted similarly to the propcess described in the study by Oskouei et al. [43].

### 2.3. Proposed Method

#### 2.3.1. Feature Extraction and Dimensionality Reduction

A DenseNet121 pretrained on ImageNet was used to extract features from individual image patches [44]. Each image patch was resized to 224×224 pixels. Extracted features were then used during the optimization of the dimensionality reduction transformation matrix with the proposed evolutionary algorithm. The presented algorithm aims to identify the optimal transformation matrix for dimensionality reduction, where the silhouette score is used as a fitness function. This measure captures the quality of clusters in the lower-dimensional space by comparing intra-cluster tightness ($a_{i}$) and inter-cluster separation ($b_{i}$):

(1) $$S=\frac{1}{n}\sum_{i=1}^{n}\frac{b_{i}-a_{i}}{\max(a_{i},b_{i})}$$

where $a_{i}$ is the intra-cluster tightness, which is the average distance between the *i*-th sample and all other points within the same cluster. It quantifies how closely the *i*-th point is grouped with its cluster neighbors—lower $a_{i}$ values indicate higher intra-cluster compactness. $b_{i}$ represents inter-cluster separation, which is the minimum average distance between the *i*-th sample and all points in any other cluster. It measures how well the *i*-th point is separated from its nearest neighboring cluster—higher values of $b_{i}$ indicate better separation between clusters.

The evolutionary algorithm iteratively generates transformation matrices over multiple generations. In each generation, the matrices that yield the highest silhouette scores are selected as the fittest, then used to produce new offspring matrices for subsequent generations. This iterative process continues until a predetermined number of generations is achieved. A high-level overview of the proposed algorithm is Algorithm 1.

In this context, the patch features extracted from the input data form the high-dimensional feature space that serves as the input to the dimensionality reduction process. Each candidate transformation matrix ($W_{i}$) operates on these patch features by mapping them into a lower-dimensional space. The quality of this mapping is then assessed by the silhouette score ($S(W_{i})$), which evaluates how well the patch features are clustered after the projection. The evolutionary algorithm iteratively refines $W_{i}$ to discover a projection that best preserves the discriminability of the patch features, yielding a lower-dimensional embedding while separating classes.

The optimized matrix was applied to all the feature vectors extracted from the patches of the whole segmented tissues to create low-dimension vectors. Using DenseNet121, patch features have a dimension of 1024, and they are transformed into two-dimensional features. For each WSI, the transformed features were stitched together, then padded or trimmed to ensure a consistent size, resulting in tensors of fixed dimensions for all WSIs. The reassembled tensor, along with spatial positions of patches, was used to construct graphs, where nodes represented the patches and edges were established based on proximity using KDTree for efficient neighbor querying [46,47].
**Algorithm 1** Evolutionary algorithm for dimensionality reduction1:**Input:** Population size *P*, generations *G*, mutation rate μ, crossover rate ρ, fitness function *S*2:**Output:** Optimized transformation matrix $W^{*}$3:**Step 1: Initialization**4:Generate an initial population of transformation matrices: $\left\{W_{1},W_{2},\ldots ,W_{P}\right\}$5:**for** g=1 to *G* **do**6:   **Step 2: Fitness Evaluation**7:   **for** each candidate $W_{i}$ in the population **do**8:          Compute fitness: $S(W_{i})$ (e.g., silhouette score)9:   **end for**10: **Step 3: Selection**11: Retain the top-performing candidates based on fitness12:    **Step 4: Crossover**13:    **for** each pair of parents $W_{{\mathrm{parent}}_{1}},W_{{\mathrm{parent}}_{2}}$ **do**14:        **if** random() <ρ **then**15:            Generate offspring:$$W_{\mathrm{child}}=\frac{W_{{\mathrm{parent}}_{1}}+W_{{\mathrm{parent}}_{2}}}{2}$$16:        **end if**17:    **end for**18:    **Step 5: Mutation**19:    **for** each offspring $W_{\mathrm{child}}$ **do**20:        **if** random() <μ **then**21:           Introduce random perturbation:$$W_{\mathrm{mutated}}=W_{\mathrm{child}}+\epsilon ,\;\epsilon ∼N\left(0,\sigma^{2}\right)$$22:        **end if**23:    **end for**24:    **Step 6: Replacement**25:    Replace the worst-performing candidates in the population with offspring26:**end for**27:**Return:** Best-performing transformation matrix $W^{*}$ based on fitness *S*

The corresponding graphs were saved in structured directories in Pickle format. These graphs were later used for training and testing of the WSI classifier model. Patch positions were stored in JSON format for the training and testing datasets. The thumbnails with annotations, including the bounding boxes around the extracted patches, were saved for inspection. The outputs were further organized into class-specific directories to enable seamless integration into machine learning workflows.

#### 2.3.2. Whole Slide Image Classification

**Figure 2:**
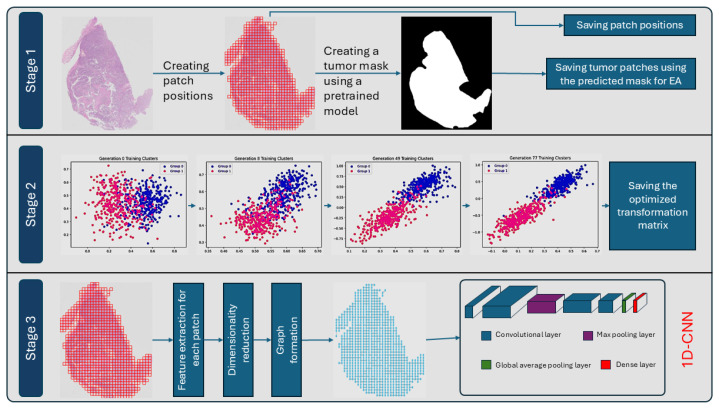
Overview of the proposed three-stage pipeline for tumor region processing and classification. **Stage 1:** Patch positions are created from the whole slide image (WSI), and a tumor mask is generated using a pretrained model. Tumor regions are identified, and patch positions and tumor patches are saved for downstream processing. **Stage 2:** An evolutionary algorithm (EA) is used to optimize a transformation matrix by iteratively clustering feature embeddings across generations, leading to better separation between the two classes of labeled data. The final transformation matrices are saved. **Stage 3:** Features are extracted from the saved patches, followed by dimensionality reduction using the saved transformation matrices and graph formation based on spatial positions. A 1-Dimensional Convolutional Neural Network (1D-CNN) is then applied for classification.

After the graphs were constructed and saved in structured directories, features from each node of each graph were stacked without spatial information and fed to a 1D-CNN for classification. The classifier processed the feature tensor to extract key patterns; aggregated them into a single representation; and, finally, outputted the class probabilities for the WSI, with class 0 referring to AC and class 1 referring to SCC. An overveiew of the proposed pipeline is presented in Figure 2.

#### 2.3.3. Model Training

To address class imbalance during training, a balanced random data generator was implemented, similar to the study by Pedersen et al. [48]. This generator ensures that each batch contains an equal number of samples from each class by employing a random sampling scheme. To ensure uniform input shapes for the CNN model, the node feature matrices were padded to a fixed size of maximum nodes in graphs. The number of nodes was dynamically determined based on the largest graph in the training data, which was saved along with the model.

The CNN classifier, a 1D convolutional neural network, includes convolutional, max-pooling, and global average pooling layers. The classifier head is a dense layer with softmax activation.

The presented model was trained using the Adam optimizer, with a learning rate of 0.001, using categorical cross-entropy as the loss function, and trained for 400 epochs with a batch size of eight. Early stopping with a patience of 20 epochs was utilized, monitoring validation loss and keeping only the best-performing model in terms of validation loss. To evaluate performance during training, the accuracy, AUC, and F_1_ score (macro-averaged) were monitored as metrics.

### 2.4. Experiments

To identify suitable hyperparameter settings for the evolutionary algorithm, we conducted a preliminary parameter search across a representative sample of candidate values. Specifically, we varied four key parameters:

- Population size: 5, 10, and 15;
- Generation limit: 1000, 5000, 15,000, and 30,000;
- Mutation rate: 0.5, 0.6, 0.7, and 0.8;
- Crossover rate: 0.4, 0.5, 0.6, and 0.7.

Each parameter combination was evaluated on a randomly sampled 10% subset of extracted patches to assess its impact on the quality and stability of the silhouette score. Using this approach, we could identify the parameters that balanced computational cost and optimization performance the best, providing a solid basis for subsequent large-scale experiments.

To place the proposed EA projector in context, we repeated the entire dimensionality reduction workflow with two standard reference techniques. For the linear baseline, we used Linear Discriminant Analysis (LDA) by applying the scikit-learn module [49]. We applied the model to the extracted DenseNet-121 features and exported the learned transformation matrix for later use on the test set. This guarantees a projection of, at most, C−1 dimensions, where *C* is the number of classes. The nonlinear comparator was the Uniform Manifold Approximation and Projection (UMAP), with default parameters of the Euclidean metric and 15 neighbors. The embedding dimensionality matched that of the proposed EA [50].

To evaluate the proposed framework, OKEN was benchmarked against a state-of-the-art Vim4Path framework on both test sets. The effectiveness of individual components was assessed through an ablation study that investigated the following aspects: backbone architecture, dimensionality reduction techniques, use of data augmentation, and the choice of classifier head. Table 2 provides an overview of the conducted experiments. After conducting these experiments, the best performing method of the OKEN framework and the models from Vim4Path were then compared for two different magnification levels (×10 and ×2.5). Magnification of ×10 was used to compare the results with those of the Vim4Path method, and ×2.5 magnification was tested to evaluate the performance of the model with a lower resolution and more global context.

In addition to the proposed 1D-CNN, our study also evaluated several alternative classifiers to benchmark performance. We implemented a 2D-CNN that treats the node feature matrix as a two-dimensional image by reshaping the data, enabling the use of standard 2D convolution and pooling operations to capture spatial correlations.

We further explored a graph convolutional neural network (GCNN) that directly incorporates the graph’s structure by propagating features through a normalized adjacency matrix, utilizing the relationships between nodes. The CGNN model is built using TensorFlow and is designed to process graphs with variably sized node sets by incorporating padded or truncated representations of node feature matrices and adjacency matrices [51]. It consists of two successive custom-defined GraphConv layers, each applying a graph convolution operation based on the normalized adjacency matrix:

(2) $$\hat{A}=D^{-1/2}\left(A+I\right)D^{-1/2}$$

where *A* is the raw adjacency matrix, *I* is identity matrix, *D* is the degree matrix, and $\hat{A}$ is the normalized adjacency matrix. These layers utilize trainable weights to transform node features, followed by ReLU activation. After the convolutional layers, the network applies a global average pooling operation over the node dimension to derive a fixed-length graph-level representation. A dense softmax-activated output layer is then used at the end for classification.

Moreover, classical machine learning approaches were employed by first aggregating node features via a simple mean operation. In this category, we compared the performance of a decision tree, a random forest, a support vector machine, and an XGBoost classifier, providing a comprehensive overview of both deep learning and traditional methods for graph-based classification tasks.

Classification was also tested using adaptedMIL and TransMIL for the transformed features [52,53]. During the training time for both MIL and TransMIL, the data loader retrieved the bag of graphs assigned to a slide, normalized every adjacency matrix, and padded variable sizes with zeros to ensure a fixed tensor size for all inputs. A masked mean over the nodes produced one fixed-length embedding per graph, and the embeddings were reshaped back to a bag tensor. For adaptation of MIL, slide-level reasoning was carried out with a gated attention mechanism in the style of Ilse et al. (2018) [52].

For adaptation of TransMIL, based on the architecture of Shao et al., a learnable classifier token was prepended, and a trainable positional embedding was added [53]. A stack of two Transformer encoder blocks modeled relationships among instances and routed contextual information to the classifier tokens.

To test the generalizability of the proposed model over various tissue qualities, scanners, and staining techniques, the model was tested on the images downloaded from TCGA datasets. To compare the performance, we also tested the TransMIL classifier in conjunction with the proposed OKEN framework and Vim4Path models on the same images.

**Table 2:** Overview of all experiments.

Framework	Backbone	Classifier	Data Refinement	Test Set
OKEN	MobileNetV2 [54]	GCNN	None	Biobank1
	EfficientNetB0 [55]			
	ResNet50 [45]			
	InceptionV3 [56]			
	DenseNet121 [44]			
	DINO-ViT-s16 [31]			
OKEN	DenseNet121	DT	None	Biobank1 HULC
		XGB		
		RF		
		SVM		
		MIL		
		TransMIL		
		GCNN		
		2D-CNN		
		1D-CNN		
OKEN	DenseNet121	1D-CNN	Aug	Biobank1 HULC *
			Aug + PCA	
			Aug + EA	
			UMAP	
			LDA	
Vim4Path	DINO-ViT-s16 [31]	CLAM	None	Biobank1 HULC
	DINO-Vim-s16 [33]			
OKEN Vim4Path	DenseNet121	1D-CNN	Aug + EA	LUAD and LUSC from TCGA
	DINO-ViT-s16	TransMIL		
	DINO-Vim-s16	CLAM		

All models are trained on Biobank1’s training set. * Only Aug + EA was tested on the external dataset. Abbreviations: ViT: Vision Transformer; Vim: Vision Mamba; MIL: Multiple-Instance Learning; TransMIL: Transformer-based Multiple-Instance Learning; PCA: principal component analysis; UMAP: uniform manifold approximation and projection; LDA: linear discriminant analysis; EA: evolutionary algorithm; Aug: augmentation; HULC: Haukeland University Hospital Lung Cancer; k-D CNN: K-dimensional convolutional neural network; GCNN: graph convolutional neural network.

### 2.5. Evaluation

To evaluate the patch-wise tumor classifier model, we assessed its performance on the HULC dataset, where segmentation results were already available. We calculated the average Dice similarity coefficient, similar to our previous study [43].

To obtain a comparison regarding the computational cost of the proposed EA method compared with LDA and principal component analysis (PCA), we calculated the runtime average of 50 generations of the EA method, along with the standard deviation. We also captured the execution time of PCA and LDA.

To compare the designs described in Table 2, three different metrics were computed: accuracy (ACC), F_1_ score, and Area Under the Curve (AUC). These were calculated for both the Biobank1 and HULC test sets. To assess statistical significance in model performance between three deep learning models (two from Vim4Path and one from OKEN) at different magnification levels, WSIs were analyzed to predict the target tumor subtype. For each WSI, the models produced probabilities for two classes, and the predicted class was determined by thresholding class 1 prediction at 0.5. The experiment was conducted for two different magnification levels of ×10 and ×2.5.

A logistic regression analysis was performed using the binary outcome as the dependent variable, with model type and magnification level as categorical predictors. Since multiple predictions were obtained from the same WSI, standard errors were clustered by sample ID to account for intra-sample correlation. The analysis was performed in Python using the statsmodels library [57]. A significance level of 5% was used to assess statistical significance.

### 2.6. Hardware and Software Configurations

Experiments were conducted on an Intel Xeon Gold 6230 @ 2.10Ghz central processing unit, using a Quadro RTX 6000 NVIDIA (24 VRAM) dedicated Graphics Processing Unit (GPU), 256 GB RAM, and a regular hard drive. OKEN was implemented in Python 3.10.12, using cuCIM v23.10.0 to process WSIs, TensorFlow v2.13.0 for model training and inference, and scikit-learn v1.5.1 for metric computation and comparison with traditional machine learning models [40,49,51]. For gradient-boosting decision trees, the XGBoost v2.1.4 library was used. Statistical analysis was performed using statsmodels [57,58]. The OKEN framework and source code to reproduce the experiments have been made openly available at https://github.com/AICAN-Research/OKEN (accessed on 20 May 2025).

### 2.7. Visualizing Transformed Features’ Geometries

To evaluate the impact of the proposed dimensionality reduction method, we began by randomly selecting image patches from 22 of the test WSIs. We extracted features from these patches using our pretrained DenseNet121 as before, generating a high-dimensional representation of each patch. Next, we applied the proposed EA-based dimensionality reduction transformation to these features. Then, we used t-distributed Stochastic Neighbor Embedding (t-SNE) and uniform manifold approximation and projection (UMAP) on the original (high-dimensional) features, as well as on the EA-transformed features [50,59].

## 3. Results

The tumor classifier model that was evaluated on the HULC dataset resulted an average Dice similarity coefficient of 0.823, with a standard deviation of 0.093 and a 95% confidence interval ranging from 0.803 to 0.843.

Throughout the hyperparameter search, no notable change was observed in performance with various population sizes; therefore, to preserve time, a population size of five was chosen. Other parameters that yielded better results were a generation limit of 30,000, a mutation rate of 0.8, and a crossover rate of 0.4.

Table 3 summarizes the performance of OKEN using various backbone architectures on the Biobank1 test set with a GCNN classifier. Among the evaluated models, DenseNet121 achieved the best ACC and F_1_ score, whereas InceptionV3 attained the highest AUC. Consequently, DenseNet121 was selected as the backbone architecture for subsequent experiments.

Table 4 summarizes the performance of the OKEN framework using DenseNet121 for feature extraction in combination with different classifiers. On the Biobank1 test set, XGB and 1D-CNN performed the best, with a similar ACC, but XGB had a higher F_1_ score and 1D-CNN had a higher AUC. The 1D-CNN generalized better to the HULC test set, performing best in terms of ACC and F_1_ score, with the GCNN performing similarly in terms of ACC and with a higher AUC. In contrast, conventional classifiers (DT, XGB, RF, and SVM) failed to generalize to the new test set (F_1_ score ≈ 0.5). Consequently, the 1D-CNN was selected as the classifier. TransMIL performed better across both datasets, demonstrating stronger results and greater generalizability than MIL.

Table 5 presents the performance of DenseNet121 as the feature extractor when paired with augmentation and dimensionality reduction methods, using the 1D-CNN classifier on the Biobank1 test set. Using the proposed evolutionary algorithm with data augmentation produced the best classification accuracy across all evaluation metrics. This approach also generalized well to the HULC test set. In contrast, using PCA with two principal components resulted in a poor silhouette score of 0.027, whereas using EA resulted in silhouette scores of 0.551 and 0.498 on the same data with and without augmentation, respectively.

Table 6 shows the performance of the proposed OKEN framework compared to Vim4Path, evaluated at two magnification levels across both test sets. The results of the logistic regression analysis are presented in Supplementary Table S1. In all experiments, the ViT-based variant of Vim4Path significantly outperformed its Vim counterpart (*p* = 0.014). Replacing Vim4Path with OKEN, using a CNN backbone, further significantly improved classification performance (*p* < 0.001). Although performance differences were observed between designs employing different magnification levels, these differences were not statistically significant (*p* = 0.338).

Table 7 summarizes the performance of the proposed model on the TCGA datasets. Table 8 shows the inference runtimes of two OKEN variants and two Vim4Path variants on a representative WSI, applied at a magnification level of ×2.5. The OKEN-DenseNet121-1D-CNN demonstrated the fastest inference runtime of 13.077 s. Both OKEN variants demonstrated faster inference runtimes compared to the corresponding Vim4Path models.

**Figure 3:**
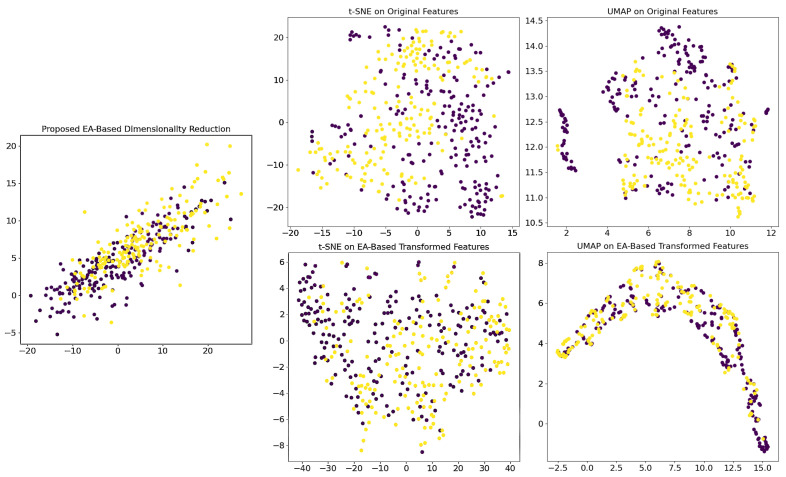
Comparison of dimensionality reduction techniques applied to high-dimensional features extracted from image patches of 22 whole slide images. The (**top-left**) plot shows the result of the proposed EA-based transformation. The remaining plots display the outputs of UMAP (**top-middle** and **top-right**) and t-SNE (**bottom-left** and **bottom-right**) applied to both the original features and the EA-based transformed features. The two visualized colors indicate the two tumor subtype classes. Abbreviations: EA: evolutionary algorithm; UMAP: Uniform manifold approximation and projection; t-SNE: t-distributed stochastic neighbor embedding.

Figure 3 compares the results of applying the UMAP and t-SNE methods to both the original features and the transformed features using the dimensionality reduction matrix.

The evolutionary optimization with the mentioned augmentation techniques on the declared hardware elements required, on average, 9.812 (SD = 0.058) seconds per generation when processing 206 images during each generation. As a result, the total runtime for 30,000 generations was roughly ∼81.8 h, yielding 150,000 candidate evaluations and processing 6,180,000 patches throughout the run. The PCA method, however, yielded the transformation matrix in 21.031 s and LDA in 19.186 s using 824 patches. There was no augmentation included in the PCA- and LDA-based dimensionality reduction.

## 4. Discussion

In this study, we developed the OKEN framework for the classification of NSCLC tumors into AC and SCC. The framework was evaluated using WSIs from internal and external lung cancer cohorts. OKEN uses a novel evolutionary algorithm-based dimensionality reduction method tested with different backbone architectures, data refinements, and classifier designs. This framework has been made publicly accessible. In our experiments, the OKEN framework with a DenseNet121 backbone and a 1D-CNN classifier—enhanced with augmentation and evolutionary optimization—achieved an F_1_ score of 0.721 on the internal test set. Other classifiers, such as XGB and GCNN, achieved comparable accuracy but demonstrated lower F_1_ scores. The 1D-CNN configuration generalized well to the external test set (F_1_ = 0.772) and had the fastest inference time among the compared methods (∼13.1 s per slide). Logistic regression analysis confirmed that the OKEN framework significantly improved performance (*p* < 0.001); however, differences due to magnification were not significant (*p* = 0.338).

The comparative experiments showed that the 1D-CNN approach outperformed the GCNN and 2D-CNN alternatives. This suggests that, in this classification task, the key information comes mainly from which features are present and how often they occur rather than from where they are located. In other words, the overall collected existence of features, no matter how they are arranged in the image, is the main factor determining the class. Thus, a more complex spatial modeling does not necessarily yield superior results in every application. This finding also highlights the importance of aligning the choice of model architecture with the characteristics of the data and the target prediction.

The observed discrepancy between high AUC/ACC and low F_1_ scores in Vim4Path models was likely due to class imbalance. Although AUC and ACC are less sensitive to imbalance, the F_1_ score, which is a harmonic average between precision and recall, drops when the minority class is underrepresented. The MIL framework used in Vim4Path struggled with imbalance because important minority-class instances were diluted among the majority class.

In the presented MIL scheme in Vim4Path, the bag-weight hyperparameter effectively balances bag-level and instance-level supervision. When the bag weight is set closer to 1.0, the model focuses more on the overall bag label, which may obscure rare positive instances. Conversely, setting it closer to 0.0 encourages the model to pay greater attention to individual instances, which can help highlight minority signals but may also increase the risk of overfitting to noise. In practice, a fixed bag weight often fails to adequately address class imbalance. One potential solution is to schedule or adaptively adjust the bag weight during training. For instance, the model can increase its focus on instances when minority-class recall is low, helping the network continuously re-balance its attention toward those scarce yet important positive instances. To further address the imbalance issue, we employed random sampling in the proposed OKEN framework to ensure class balance within each mini-batch, resulting in improved F_1_ scores.

We also conducted experiments with MIL and TransMIL, along with OKEN. Although the simple MIL model relies on instance-level attention and pooling, TransMIL achieved substantially better performance in our experiments, even after only a basic hyperparameter search. Unlike traditional MIL, TransMIL explicitly models correlations among instances within each bag through a Transformer-based self-attention mechanism. By learning morphological relationships across patches, it acquires richer contextual information than standard MIL.

A study by Chen et al. introduced an annotation-free whole-slide training method that achieved high AUC values for AC and SCC classification, demonstrating the potential of training neural networks on entire WSIs without detailed annotations [60]. This paper proposed a WSI training approach using unified memory and GPU memory optimization techniques, enabling standard CNNs to handle extremely large image inputs without altering model architectures or training pipelines. Furthermore, the authors proposed the use of slide-level labels only, thereby avoiding more granular annotations [60]. In this sense, their approach is similar to the methods tested in our study. Similar to their method, our method does not rely on detailed annotations; however, it requires a tumor/non-tumor patch classifier to extract relevant patches, which are subsequently used in the EA to find the optimal dimensionality reduction matrix.

Wong and Yi conducted a comprehensive evaluation of pre-trained feature extractors within MIL frameworks for WSI classification, particularly focusing on NSCLC subtypes [61]. They assessed the impact of various pre-training datasets (ImageNet-1K vs. ImageNet-21K), backbone architectures (ResNet50, ConvNeXt-B, ViT-S/16, and Swin-B), and pretraining methods (supervised vs. self-supervised learning) on the performance of four state-of-the-art MIL models: ABMIL, DSMIL, TransMIL, and DTFD-MIL [52,53,62,63]. Their findings indicate that models utilizing larger and more diverse pre-training datasets, modern and deeper backbones, and self-supervised learning methods, particularly DINO with ViT backbones, achieved superior classification performance on the TCGA-NSCLC and Camelyon16 datasets [61].

Vim4Path uses a DINO-based feature extractor that is pretrained on external data and was used in our experiments without fine-tuning. Although pretrained models capture general visual patterns, they may not be perfectly suitable for specific features of our dataset, which could explain the relatively poorer results observed for Vim4Path in this study. OKEN, however, applies an evolutionary algorithm to transform the features to a lower dimension space before training, effectively “fine-tuning” them for our domain. This domain-specific adaptation, optimizing for clustering quality (as measured by the silhouette score), explains the improved performance over PCA’s linear approach.

Although model performance must still be improved for clinical utility, OKEN performed better than other tested dimension-reduction techniques. The explanation for this could be that OKEN directly exploits the found augmented labeled data to optimize the feature transformation matrix in a larger space instead of depending on generic objectives such as variance preservation or neighborhood reconstruction. By taking the silhouette score with known class assignments as the objective to optimize, OKEN can learn mappings that form the input data into a shape in which samples from the same class are pulled together but pushed away from the samples with different labels. This is highly relevant to the classification task.

In contrast to linear methods such as LDA, which impose a fixed parametric form and use only a single subspace of candidate solutions, OKEN uses an evolutionary learning paradigm with augmented data in each generation. This enables exploration of a much richer space of candidate transformations. Only mappings that consistently produce high supervised silhouette scores under adversarial noise and sampling are retained; therefore, overfitting can be mitigated. The proposed method can also take advantage of nonlinear parameters.

The proposed evolutionary algorithm is computationally more demanding than traditional dimensionality reduction methods such as PCA or LDA. However, its improved quality may justify the computational cost in applications where precision and reliability are more important.

Overall, Vim was more prone to performance degradation than ViT, particularly when the feature extractor was not fine-tuned. Vim inherently encodes spatial and sequential dependencies due to its recurrent state-space structure. This design can introduce an inductive bias towards local contextual relationships. If features related to our data differ from the pretraining dataset (Camelyon16 [64]), Vim’s representations may quickly become suboptimal because the learned local sequential patterns seem to be specific to the distribution of the dataset’s that was used for pretraining. This might be the case for our test sets.

ViT’s representations, on the other hand, are comparatively more general, since ViT does not inherently encode strong local sequence patterns. It treats each patch independently and, hence, generalizes better, providing robustness when the domain shifts slightly. ViTs, being more general-purpose and less reliant on strong inductive biases, can adapt better to slightly different distributions without fine-tuning. Hence, they can benefit from generalized patterns learned through global attention mechanisms.

Compared to classical machine learning methods (DT, RF, SVM, and XGB), which show competitive performance internally but fail to generalize to external data, deep learning models like CNNs and GCNNs learn hierarchical spatial and relational features that are more robust to dataset shifts. Classical methods often overfit the training data because they rely on fixed feature representations, whereas CNNs benefit from weight sharing and translation invariance.

In the external test set, the level of differentiation and the presence or absence of necrosis were recorded for all tumors. Differentiation was classified as well (moderately, poorly, or undifferentiated). The external test set included few well differentiated tumors, accounting for 13% of all ACs and 4% of all SCCs. Necrosis was present in a majority of tumors, occurring in over 60% of ACs and more than 90% of SCCs. Data on differentiation grade and necrosis were not available for the internal test set, but differences in morphology between the two test sets may explain the discrepant results.

Logistic regression with cluster-robust standard errors confirmed that both the Vim4Path-ViT-S-16 and OKEN-DenseNet121 architectures increase the odds of correct classification compared to a baseline model and that, within our tested range, magnification did not significantly affect performance. However, the relatively small sample size and limited magnification range suggest that further validation is needed.

Although higher magnifications can present more cellular details, lower magnification may be more adept at rendering an overview of tissue morphology that may also be necessary for correct classification. This may explain the statistical indifference in the results between high and low magnification.

The EA-based transformation is specifically engineered to maximize the silhouette score by compressing intra-class distances and expanding inter-class distances, thereby creating a feature space where classes are compact and well-separated. Applying UMAP to these already 2D-transformed features (Figure 3) further refines this inherent structure by preserving local topological relationships, resulting in even narrower and more concentrated clusters, highlighting the reduced within-class variance. Although UMAP is typically employed for to reduce high-dimensional data, in this context, its role shifts to fine-tuning the existing structure rather than reducing dimensions, as its non-linear optimization re-scales distances to better reflect the underlying manifold. This enhanced clustering effect indicates that the EA-based method reduced the intra-class variance. In contrast, t-SNE, which focuses on preserving local pairwise similarities by minimizing the Kullback–Leibler divergence, tends to exaggerate the separation between clusters to avoid crowding and is sensitive to parameters like perplexity. As a result, even though the EA transformation produces compact clusters, t-SNE does not display the same level of narrow distribution because its optimization approach does not further tighten the clusters in the same manner as UMAP. To verify that the observed outcomes are not contingent upon specific patch features, we evaluated multiple randomly selected WSIs and patches, all of which yielded consistently analogous results.

The OKEN framework shows promise, particularly in its performance gains, when the dimensionality reduction component is carefully managed to minimize bias in the training data. Although reducing the output dimensions to a very low number may result in improved silhouette scores, this approach risks omitting critical information. Thus, this also presents a challenge in ensuring that essential data is retained. We used augmentation methods to improve generalizability and conducted a test to evaluate the performance on a number of randomly selected images from the external dataset (TCGA). Results suggest that the proposed model may be relatively robust to variance in scanner or staining techniques.

In this work, the silhouette score was used as the fitness measure for its straightforward assessment of intra-cluster cohesion and inter-cluster separation. There are other metrics that can measure clustering quality, such as the Davies–Bouldin Index (DBI) and the Fisher ratio [65,66]. DBI measures the average similarity between each cluster and its most similar neighbor, with lower values indicating better separation, and operates at the cluster level rather than the sample level. The Fisher ratio assesses the ratio of between-class to within-class variance, highlighting class discriminability. Future work could explore such alternative metrics individually or in combination.

A weakness of the proposed method is the computational expense associated with the evolutionary algorithm that optimizes the dimensionality reduction. This process can become particularly time-consuming with larger datasets or when augmentation methods are applied. Furthermore, a vulnerability is related to the tumor segmentation model; its performance is pivotal, since the precise and meaningful selection of tumor patches plays an essential role in the overall optimization strategy.

We did not systematically assess how silhouette scores relate to classification metrics. Such an analysis could clarify the links between cluster separation and predictive performance. Future work could explore this further. Likewise, examining how the reduced feature size affects the results is important, as dimensionality reduction can impact clustering and classification, and selecting the optimal number of components often requires extensive testing or cross-validation.

In the future, to explain which parts of the input are more influential on the model’s outcome, we aim to improve interpretability by incorporating methods tailored to each model architecture. For the 1D-CNN, feature attribution methods can estimate the contribution of each input feature to the prediction. In the 2D-CNN, spatial attention techniques such as Grad-CAM can highlight discriminative regions in the input patches. For the GCNN, node-importance measures like attention weights, centrality indices, or gradient-based attribution can help identify influential nodes and edges in the graph.

Moreover, even though the dimensionality of the latent space is configured manually in our method, adopting dynamic or adaptive strategies such as empirical dimension sweeping could improve robustness. Although this aspect was beyond the scope of the current study, we see this as a promising avenue for future work.

The proposed evolutionary optimization method provides substantial performance gains but remains computationally intensive. Several strategies could mitigate this cost. Parallelization across multiple GPUs or high-performance clusters can significantly reduce the time required. Early stopping or convergence detection could limit the number of necessary generations. Additionally, surrogate fitness modeling or low-fidelity approximations may improve efficiency, which can be investigated in future work.

Furthermore, the current version of our model does not incorporate mechanisms for interpretability or visualization of salient regions, such as attention maps or saliency techniques. Integrating such approaches would enhance the transparency and explainability of the predictions, which is particularly valuable in clinical applications. We consider this an important direction for future work.

## 5. Conclusions

In summary, our study shows that the proposed OKEN framework effectively enables WSI classification of non-small cell lung cancer. It applies an evolutionary algorithm to optimize dimensionality reduction while preserving class separability. OKEN not only achieves competitive classification performance and generalizability but also maintains a relatively fast and light computational profile. Furthermore, exploring explainability, particularly with the GCNN classifier to identify key contributing regions, is also a valuable direction for future work.

## Figures and Tables

**Table 1:** Histological subtype distribution in the datasets.

Subtype	Biobank1—Training and Validation	Biobank1—Test	HULC—Test
AC	131	22	45
SCC	60	8	41
Total	191	30	86

Abbreviations: AC: adenocarcinoma; SCC: squamous cell carcinoma; HULC: Haukeland University Lung Cancer.

**Table 3:** OKEN performance with different backbones using the GCNN classifier, evaluated on the Biobank1 test set. The best performance in each category is indicated in bold.

Backbone	Biobank1		
	ACC	AUC	F_1_-Score
MobileNetV2	0.566	0.569	0.466
EfficientNetB0	0.566	0.593	0.422
ResNet50	0.500	0.583	0.494
**InceptionV3 **	0.699	**0.748**	0.600
**DenseNet121**	**0.733**	0.707	**0.700**
DINO-ViT-s16	0.566	0.613	0.494

Abbreviations: ACC: accuracy; AUC: area under the curve.

**Table 4:** OKEN performance with DenseNet121 feature extraction and various classifiers for Biobank1 and HULC test sets. The best performance in each category is indicated in bold.

Classifier	Biobank1			HULC		
	ACC	AUC	F_1_-Score	ACC	AUC	F_1_-Score
DT	0.733	0.627	0.619	0.581	0.575	0.552
**XGB**	**0.833**	0.784	**0.754**	0.558	0.575	0.527
RF	0.766	0.801	0.610	0.511	0.606	0.470
SVM	0.733	0.625	0.423	0.511	0.630	0.338
MIL	0.699	0.792	0.600	0.593	0.647	0.593
TransMIL	0.700	0.727	0.653	**0.709**	**0.722**	0.708
**GCNN**	0.733	0.707	0.700	0.686	0.707	0.686
2D-CNN	0.666	0.737	0.625	0.558	0.636	0.527
**1D-CNN**	**0.833**	**0.875**	0.721	0.686	0.688	**0.772**

Abbreviations: DT: decision tree; XGB: extreme gradient boosting; RF: random forest; SVM: support vector machine; MIL: multiple-instance learning; TransMIL: transformer-based multiple-instance learning; GCNN: graph convolutional neural network; kD-CNN: K-dimensional convolutional neural network; HULC: Haukeland University Lung Cancer; ACC: accuracy; AUC: area under the curve.

**Table 5:** OKEN performance with DenseNet121 backbone, 1D-CNN classifier, and different data refinement techniques, evaluated on the Biobank1 test set. The best performance in each category is indicated in bold.

Data Refinement	Biobank1			HULC		
	ACC	AUC	F_1_-Score	ACC	AUC	F_1_-Score
None	0.633	0.686	0.612	–	–	–
Aug	0.666	0.691	0.652	–	–	–
PCA	0.733	0.705	0.423	–	–	–
UMAP	0.733	0.742	0.423	–	–	–
LDA	0.699	0.611	0.653	–	–	–
**Aug + EA **	**0.833 **	**0.875**	**0.721**	0.686	0.688	0.772

Abbreviations: Aug: data augmentation; PCA: principal component analysis; EA: evolutionary algorithm; HULC: Haukeland University Lung Cancer; ACC: accuracy; AUC: area under the curve.

**Table 6:** Performance metrics for different methods on the Biobank1 and HULC test sets. The best performance in each category is indicated in bold.

Method	Biobank1			HULC		
	ACC	AUC	F_1_-Score	ACC	AUC	F_1_-Score
**Vim4Path-ViT-S-16 (×2.5)**	0.700	0.693	0.470	**0.732**	**0.841**	0.684
Vim4Path-ViT-S-16 (×2.5)	0.700	0.642	0.470	0.581	0.793	0.250
Vim4Path-Vim-S-16 (×10)	0.733	0.721	0.555	0.488	0.607	0.620
Vim4Path-Vim-S-16 (×2.5)	0.700	0.625	0.526	0.511	0.533	0.086
OKEN-DenseNet121 (×10)	0.733	0.836	0.582	0.709	0.837	0.695
**OKEN-DenseNet121 (×2.5)**	**0.833**	**0.875**	**0.721**	0.686	0.688	**0.772**

Abbreviations: ViT: Vision Transformer; Vim: Vision Mamba; S: small; HULC: Haukeland University Lung Cancer; ACC: accuracy; AUC: area under the curve.

**Table 7:** Performance metrics for different methods tested on the 48 images randomly selected from LUAD and LUSC from TCGA. The best performance in each category is indicated in bold.

Method	ACC	AUC	F_1_-Score
**OKEN-DenseNet121-1D-CNN**	0.708	**0.809**	0.704
**OKEN-DenseNet121-TransMIL**	**0.714**	0.749	**0.714**
Vim4Path-ViT-S-16 (×10)	0.489	0.670	0.329
Vim4Path-Vim-S-16 (×10)	0.595	0.608	0.612

Abbreviations: CNN: convolutional neural network; ViT: Vision Transformer; Vim: Vision Mamba; S: small; ACC: accuracy; AUC: area under the curve.

**Table 8:** Inference runtime comparison on a random representative whole slide image. The test was executed eleven times, with the first run being a warm-up run ignored in metric computation. The runtime includes patch/position creation, feature extraction, and final classification at ×2.5 magnification. The best performance is indicated in bold text.

Method	Inference Runtime (s)
Vim4Path-ViT-S-16	15.468 ± 0.133
Vim4Path-Vim-S-16	18.452 ± 0.112
OKEN-DenseNet121-GCNN	14.969 ± 0.022
**OKEN-DenseNet121-1D-CNN**	**13.077** ± **0.063**

Abbreviations: ViT: Vision Transformer; Vim: Vision Mamba; S: small; GCNN: graph convolutional neural network; CNN: convolutional neural network.

## Data Availability

The datasets generated and/or analyzed during the current study are not publicly available due to the sensitive nature of personal medical data from patients who may still be alive but might be available from Professor Hanne Sorger upon request on a mutually collaborative basis.

## Supplementary Materials

The following supporting information can be downloaded at: https://www.mdpi.com/article/10.3390/bioengineering12070733/s1, Table S1: Logistic Regression Results.

## Abbreviations

The following abbreviations are used in this manuscript:

ACC	Accuracy
AUC	Area under the curve
CLAM	Clustering-constrained attention multiple-instance learning
CNN	Convolutional neural network
DT	Decision tree
EA	Evolutionary algorithm
GCNN	Graph convolutional neural network
GPU	Graphics processing unit
HE	Hematoxylin and eosin
HULC	Haukeland University Lung Cancer
MIL	Multiple-instance learning
NSCLC	Non-small cell lung cancer
PCA	Principal component analysis
RF	Random forest
SVM	Support vector machine
t-SNE	t-distributed stochastic neighbor embedding
UMAP	Uniform manifold approximation and projection
Vim	Vision Mamba
ViT	Vision Transformer
WSI	Whole slide image
XGB	Extreme gradient boosting
