# Supplementary material for: OKEN: A Supervised Evolutionary Optimizable Dimensionality Reduction Framework for Whole Slide Image Classification
Source: Bioengineering (Basel). 2025 Jul 4;12(7):733. doi: 10.3390/bioengineering12070733 (PMC12292405; doi:10.3390/bioengineering12070733)
Supplement: Supplementary file 1 [file bioengineering-12-00733-s001.zip › bioengineering-3682285-supplementary.pdf]

Article

Supplementary Materials for: OKEN: A Supervised Evolutionary Optimizable Dimensionality Reduction Framework For Whole Slide Image Classification

Supplementary Materials

Table S1. Logistic Regression Results

Term	Coef	Std. Err	z	*p*-value	CI Lower	CI Upper
Intercept	0.3024	**0.156**	1.938	0.053	-0.003	0.608
C(model_type)[T.1]	0.4752	0.193	2.467	0.014	0.098	0.853
C(model_type)[T.2]	**0.6982**	0.198	**3.534**	**0.000**	0.311	1.085
C(Level)[T.4]	-0.1540	0.161	-0.958	0.338	-0.469	0.161
Model statistics:						
$N = 696$, Log-Likelihood = -444.86, LL-Null = -452.09						
Pseudo $R^2 = 0.01599$, LLR p-value = 0.002348						

The magnification baseline for this analysis is $10\times$. Patch Level 4 corresponds to a magnification of $2.5\times$. Coefficients represent the change in log-odds of a correct prediction relative to reference categories. Model type baseline: Vim4Path-ViT-S-16; Model type 1: Vim4Path-Vim-S-16; Model type 2: OKEN-DenseNet121; LL: Log-Likelihood; LLR: Likelihood Ratio Test statistic.
